# Beyond the control of the care home: A meta‐ethnography of qualitative studies of Infection Prevention and Control in residential and nursing homes for older people

**DOI:** 10.1111/hex.13349

**Published:** 2021-08-21

**Authors:** Gavin Daker‐White, Maria Panagioti, Sally Giles, Thomas Blakeman, Victoria Moore, Alex Hall, Paul P. Jones, Oliver Wright, Bethany Shears, Natasha Tyler, Stephen Campbell

**Affiliations:** ^1^ Division of Population Health, Health Services Research and Primary Care, Centre for Primary Care and Health Services Research The University of Manchester Manchester UK; ^2^ Division of Population Health, Health Services Research and Primary Care, NIHR Greater Manchester Patient Safety Translational Research Centre, Manchester Academic Health Science Centre The University of Manchester Manchester UK; ^3^ The University of Manchester Law School Manchester UK; ^4^ Division of Nursing, Midwifery and Social Work The University of Manchester Manchester UK

**Keywords:** Infection Prevention and Control, long‐term care facilities, meta‐synthesis, older people, qualitative studies

## Abstract

**Objective:**

This study aimed to develop interpretive insights concerning Infection Prevention and Control (IPC) in care homes for older people.

**Design:**

This study had a meta‐ethnography design.

**Data Sources:**

Six bibliographic databases were searched from inception to May 2020 to identify the relevant literature.

**Review Methods:**

A meta‐ethnography was performed.

**Results:**

Searches yielded 652 records; 15 were included. Findings were categorized into groups: The difficulties of enacting IPC measures in the care home environment; workload as an impediment to IPC practice; the tension between IPC and quality of life for care home residents; and problems dealing with medical services located outside the facility including diagnostics, general practice and pharmacy. Infection was revealed as something seen to lie ‘outside’ the control of the care home, whether according to origins or control measures. This could help explain the reported variability in IPC practice. Facilitators to IPC uptake involved repetitive training and professional development, although such opportunities can be constrained by the ways in which services are organized and delivered.

**Conclusions:**

Significant challenges were revealed in implementing IPC in care homes including staffing skills, education, workloads and work routines. These challenges cannot be properly addressed without resolving the tension between the objectives of maintaining resident quality of life while enacting IPC practice. Repetitive staff training and professional development with parallel organisational improvements have prospects to enhance IPC uptake in residential and nursing homes.

**Patient or Public Contribution:**

A carer of an older person joined study team meetings and was involved in writing a lay summary of the study findings.

## 1 INTRODUCTION

### 1.1 Getting started—Rationale for the research

During the first wave of the global coronavirus disease 2019 (Covid‐19) pandemic, the virus may have been responsible for around half of all deaths in nursing or residential homes in European countries.^1^ In England, it has been suggested that government policy privileging safeguarding the NHS and hospital discharge practices are possible reasons for the high number of deaths.^2^ However, there was less discussion about mechanisms internal to care homes that contribute to the devastating impact of the Covid‐19 pandemic, such as staff knowledge and resident behaviour, although a shortage of personal protective equipment, such as masks and gowns, was indicated.^3^ It is therefore important to understand the factors that might promote or hinder the spread of an infectious disease like Covid‐19 into and within care homes for older people.

### 1.2 Getting started—Context for the research

Previous studies have examined staff adherence to Infection Prevention and Control (IPC) guidelines, mainly looking at self‐reported behaviour through questionnaire surveys. Hand hygiene is one of the most basic strategies in IPC, and a cross‐sectional study of compliance in nursing homes found that immediate access to disinfectant materials and role modelling by senior nursing staff were important factors for successful implementation.^4^ Other approaches have also been proposed, including national initiatives, such as the use of inspection regimes or specialist infection control nurses.^5^, ^6^ Most of these interventions, as well as the bulk of relevant observational studies, had taken place in the United States.

A recent questionnaire study of nursing home staff in Italy found ambivalence and low uptake of influenza vaccination, with 34% of respondents expressing safety concerns.^7^ A similar survey in France also found ‘hesitancy’ around influenza vaccination and recommended ‘communication interventions’ to improve staff uptake.^8^ Pilot searches revealed that such issues had also been explored in more depth in at least one qualitative study, with issues around education and workload highlighted.^9^

Staff education and training has been recommended to improve IPC in care homes, although this can be challenging, given high rates of staff turnover.^10^ A recent systematic review of the effectiveness of IPC programmes in long‐term care facilities by the World Health Organisation (WHO found that monitoring and feedback, in addition to staff education, had also been used, although efforts needed to focus on at least four elements of WHO's strategy (IPC Programmes, Guidelines, Training and Hospital‐Acquired Infection surveillance)^11^ to control infections.^12^ A multimodal approach to improve hand hygiene and use of gloves noted the utility of training packages being contextualized in everyday practice.^13^ A Swedish study that set out to examine care home staff knowledge and adherence to guidelines appeared to be hampered by the fact that carriage of bacteria, and thus experience of IPC, was very limited.^14^ Moreover, the issues facing care homes in respect of transmissible infections are considerable, especially at the interface with hospitals. Infection is easily transmissible within a shared residential environment, and care homes may readily become reservoirs of hospital‐acquired infections.^15^

Similar findings in relation to IPC in residential and nursing care homes have been noted in the United Kingdom. A UK Health Protection Agency^16^ report concerning the management of *Clostridium difficile* in care homes was based on a questionnaire survey of care homes in Sussex. This survey found that many homes did not follow infection control guidance current at that time. Accordingly, recommendations were made around training, infection control management and associated standards for commissioners and inspectors of services.

### 1.3 Focus of the meta‐ethnography

A metasynthesis of qualitative studies in IPC in nursing and residential care homes for older people was conducted. Meta‐ethnography was chosen as a review and synthesis method, as it offers the opportunity to develop conceptual insights that go beyond the findings of qualitative studies.^17^ The method is akin to a systematic review in quantitative effectiveness studies, although the way in which findings are brought together is more like primary qualitative research in the way that concepts, metaphors or findings^17^ used by authors of original studies are systematically organized and compared.

Our aim was to develop interpretive insights into the factors that influence infection transmission in residents of care homes for older people. To achieve this, we set out to identify qualitative studies that would reflect the ways in which IPC is managed in care homes in practice and extract findings that yield insights into the enactment of IPC practices such as isolation, hand washing, environmental cleaning and antimicrobial management. Ethnographic and participant observation studies offer the potential to yield insights into actual (rather than self‐reported) behaviour and advance current IPC understanding that is mostly based on self‐reported data. Interview or focus group studies around knowledge, perceptions or adherence to IPC guidelines could help form hypotheses about how infection transmission might be either enabled or prevented.

## 2 METHODS

This study report has been structured according to a framework for reporting standards for meta‐ethnographies in health research.^18^ As originally described by Noblit and Hare,^17^ meta‐ethnography is a seven‐step process.^16^ These steps can be understood as approximately commensurate with the equivalent stages of a systematic review of quantitative studies, to wit: (i) ‘getting started’ (formulate a review question), (ii) ‘deciding what is relevant to the initial interest’ (develop protocol, conduct searches, select studies), (iii) ‘reading the studies’ (assess study quality, extract data), (iv) ‘determine how the studies are related’ (analyse and summarize study findings), (v) ‘translating the studies into one another’ and (vi) ‘synthesizing translations’ (meta‐analysis—where undertaken) and (vii) ‘expressing the synthesis’ (interpret results). In this report, however, we use the subheadings recommended by France et al.^18^ for reporting the results.

### 2.1 Search strategy

The bibliographic databases Medline, Embase, PsychINFO, CINAHL and ASSIA were searched from inception to May 2020 using a strategy with three modified blocks of terms (Mesh terms and keywords) derived from previously published reviews: Care homes for older people,^19^ infections (IPC focus)^20^ and some simple keywords found to have high utility in identifying reports of qualitative studies.^21^ Searches are provided in Table S1A. A number of ad hoc searches were run in Google Scholar, which is considered a good source for identifying grey literature, such as unpublished theses and dissertations.

### 2.2 Eligibility criteria

We included published reports of studies that fulfilled the following criteria:

1. Participants/setting: Involved residents, staff members or managers of nursing or residential homes for people aged over 60.
2. Studies design: Used qualitative methods of data collection (i.e., focus groups, interviews, observations) and analysis. Mixed‐methods reports were included so long as there was presentation of a thematic analysis, or similar, at some point in the publication.
3. Outcome: Focused on IPC practices such as (but not limited to) isolation, hand washing, environmental cleaning and antimicrobial management.
4. Were written in English.

### 2.3 Study selection

Titles and abstracts were independently double screened for 8% of the results (*n* = 50) by G. D. W. and S. G., with both agreeing which articles would be included. After establishing this high level of agreement, the first author completed the rest of the title/abstract screening. The full‐text screening and data extraction were shared between each coauthor, although the first author completed around 30%, purposefully selecting studies concerning different topics.

Data extraction was completed using a modified version of a previous form used in a meta‐synthesis of qualitative studies of patient safety in primary care^22^ (see Table S1B). The quality of the included studies was assessed using five fundamental criteria for reporting quality in studies for a meta‐synthesis.^23^

In most instances, text was copied and pasted from the articles into the data extraction forms, making it harder for primary data and findings to get lost in translation. Each coauthor was assigned at least one study to complete full data extraction and quality assessment.

### 2.4 Process for determining how the studies were related

The first author read the completed data extraction forms, having previously read the full texts (including the dissertation and thesis) in full, and looked for common issues or theme groups. First, the studies were divided into infection type (e.g., urinary tract infections and antimicrobials; methicillin‐resistant *Staphylococcus aureus*; scabies and ‘General Focus’), and tables of evolving theme groups were constructed. During this process, primary quotations from the studies were retained. At all stages of the translation process, groups of studies were analysed chronologically, beginning with the earliest published in each subset. Throughout the study, draft findings were circulated around the study team by the principal worker (first author) and discussed in weekly meetings to agree next steps.

### 2.5 Process of translating studies

Comparison of these infection‐specific frameworks of findings showed no differences, that is, they all spoke to common issues, e.g. around staff workload or relationships with health services. Accordingly, the articles were treated as a whole and a new framework was developed incorporating all studies. At each stage in the process, the translations were circulated to the wider team to garner alternative interpretations of meaning, significance or coherence of presentation.

### 2.6 Synthesis process

As new iterations brought findings together in different groups, their comparison was used to develop second‐order explanations by either: (i) comparing refutational data within each row of each table or (ii) determining concepts or metaphors that described the contents of the cell or row. In some cases, second‐order interpretations were found in the primary study reports, although they are not always found in descriptive studies.^24^ The second‐order interpretations (whether developed or reported) were themselves compared in each row of the Tables S2–S5 and used to form synthetic interpretations. In the tables, synthetic interpretations are shown in blue text.

### 2.7 Patient and public involvement

One experienced public contributor, who is an informal carer, attended our weekly research team meetings and contributed to discussions about refining research questions, searching and selecting studies and synthesizing the relevant data. Together with the first author, the public contributor coproduced a lay summary of the findings and advised authors on the interpretation and dissemination of results.

## 3 RESULTS

**Figure 1:**
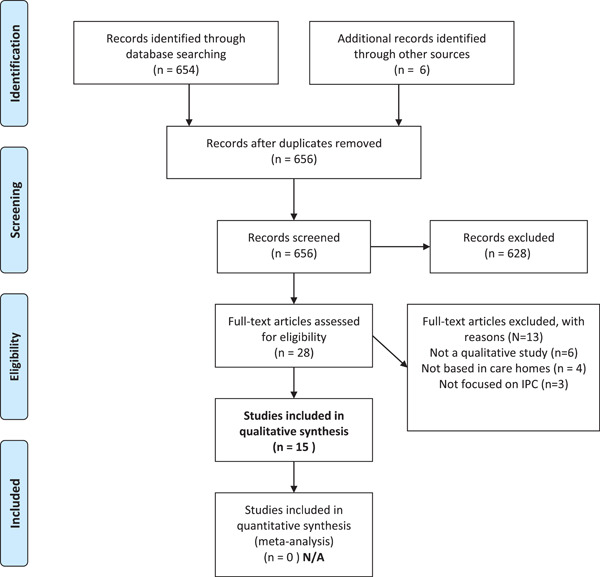
PRISMA chart

Of 656 records screened, 28 full‐text articles were initially included and assessed. A further 13 were excluded at full assessment, leaving 15 articles (including 13 unique studies because one study was reported in three different articles) eligible for inclusion (the PRISMA flowchart of the study selection process is presented in Figure 1).

### 3.1 Characteristics of included studies

All included articles were published between 2007 and 2020. Most of the studies used semi‐structured interview or focus groups, were descriptive in nature and used thematic or content analysis (Table 1). Two studies, one of which was a doctoral dissertation, included observation of IPC behaviours in staff and residents. Articles reported studies mainly undertaken in North America (*n* = 8 studies from 10 articles), with others situated in the United Kingdom (*n* = 3), South Korea and Australia. The participants of the studies were usually nursing or care home staff, but some studies also recruited residents, administrators, leads of nursing or care home facilities and health professionals. It was noteworthy that most of the included articles did not show the demographic characteristics of study participants.

**Table 1:** Features of the included articles (note that articles 6, 7 and 8 report findings from the same study)

ID	Year Pub'n	Title	Location	Study population	Data collection method	Data analysis method	Quality assessment
1^25^	2007	‘Evidence‐Based Clinical Pathways To Manage Urinary Tract Infections in Long‐Term Care Facilities: A Qualitative Case Study Describing Administrator and Nursing Staff Views’	USA and Canada	Administrators (19) and nurses (52) working in care homes enrolled in a randomized‐controlled trial	Interviews and focus groups	Thematic, descriptive	Acceptable
2^26^	2010	‘Impact of NHAP Guideline	USA	Liaison nurses (9), assistant/directors of nursing (18), clinical directors and development staff (4)	NB part qualitative: semi‐structured interviews	Content analysis, descriptive	Acceptable
		Implementation Intervention on Staff and Resident Vaccination Rates’					
3^27^	2011	‘“Somebody else's problem”? Staff perceptions of the sources and control of methicillin‐resistant *Staphylococcus aureus*’	UK	44 Hospital and 53 care home staff	Part qualitative: Focus groups as part of a mixed‐methods study	Content analysis through the lens of causal attribution theory	Acceptable
4^28^	2012	‘Infection control and meticillin‐resistant *Staphylococcus aureus* decolonisation: the perspective of nursing home staff’	UK	39 Nursing home staff	Interviews (care home managers) and focus groups (nursing home staff)	Thematic, descriptive	Acceptable
5^29^	2014	‘Antibiotic prescribing practice in residential aged care facilities‐health care providers' perspectives’	Australia	40 Nurses, 15 general practitioners and 6 pharmacists associated with 12 residential care homes	Interviews, focus groups and onsite observations	Thematic framework analysis	Acceptable
6^30^	2015	‘Infection prevention and control in nursing homes: a qualitative study of decision‐making regarding isolation‐based practices’	USA	73 Staff associated with 10 nursing homes	Semi‐structured interviews	Content analysis, based on key words search	Acceptable
7^31^	2015	‘Understanding infection prevention and control in nursing homes: A qualitative study’	USA	73 Staff working in 10 nursing homes	Semi‐structured interviews	Thematic, descriptive	Acceptable
8^9^	2015	‘Perceived barriers to infection prevention and control for nursing home certified nursing assistants’	USA	73 Staff working with 10 nursing homes	Semi‐structured interviews	Content analysis, descriptive	Acceptable
9^32^	2015	‘Scabies outbreaks in residential care homes: factors associated with late recognition, burden and impact. A mixed methods study in England’	UK	Prospective mixed‐methods study of outbreaks in 7 care homes	Part qualitative: Field notes and open‐ended questions	Thematic, descriptive	Acceptable
10^33^	2016	‘Perceptions of Gown and Glove Use to Prevent Methicillin‐resistant *Staphylococcus aureus* Transmission in Nursing Homes’	USA	19 Care staff, 7 administrators and 5 residents	Focus groups	Content analysis, descriptive	Acceptable
11^34^	2017	‘Testing the Effect of a Resident‐Focused Hand Hygiene Intervention in a Long‐Term Care Facility: A Mixed Methods Feasibility Study’	USA	6 Residents and 6 staff, purposefully sampled for a 50:50 gender split	Observation and semi‐structured interviews	Coding using Health Belief Model	Excellent
12^6^	2017	‘A national collaborative approach to reduce catheter‐associated urinary tract infections in nursing homes: A qualitative assessment’	USA	8 Organisational and 8 nursing home facility leads	Structured interviews	‘Rapid’ thematic analysis	Borderline Unacceptable
13^35^	2018	‘Nurses' Views on Infection Control in Long‐Term Care Facilities in South Korea: A Focus Group Study’	South Korea	15 Nursing staff in 5 care homes	Focus groups	Content analysis, descriptive	Acceptable
14^36^	2019	‘A Qualitative Study on Perceived Barriers and Facilitators of Implementing an Antimicrobial Stewardship Intervention in the Management of Urinary Tract Infections in a Long‐Term Care Setting’	Canada	16 Staff members	Focus groups and semi‐structured interviews	Content analysis, descriptive	Acceptable
15^37^	2020	‘Environmental service workers as potential designers of infection control policy in long‐term care settings’	USA	40 Infection, nursing and environmental service staff	Telephone and face‐to‐face interviews	Constant comparative method	Acceptable

The studies were mainly concerned with types of infections such as methicillin‐resistant *Staphylococcus aureus*,^27^, ^28^, ^33^ *C. difficile*, urinary tract Infections^6^, ^25^ and scabies.^32^ Some were focused on specific IPC practices (such as isolation,^30^ vaccination,^26^ antimicrobial management,^29^, ^36^ gown and glove use^33^ or hand hygiene^34^). Others had a more generic focus on IPC practice.^9^, ^31^, ^35^, ^37^

They were broadly acceptable at quality of reporting assessment, with one considered excellent^34^ and one borderline unacceptable.^6^

### 3.2 Outcome of relating studies or study translation

Iterative reading and reorganisation of study findings eventually yielded three coherent theme groups focused on staff motivations and behaviour; the organisation of nursing or residential care homes; and interface with other health care services. Around 50% of the data and findings centred on an essential tension between staff knowledge, behaviour and attitudes set against the challenges of workload and shift patterns (Table S2). Other groups of findings were mainly focused on the barriers and facilitators to enacting IPC in care homes at the individual staff behaviour level (Table S3); the operationalization of IPC in a shared home environment, including resident perceptions (Table S4); and issues at the interface with medical services (Table S5).

To illustrate the process by which findings were analysed to generate interpretive insights, Table 2 represents an abridged version of Table S5. The left‐hand column of the table, with the heading ‘descriptive data and participant quotes’, contains original (or raw) interview data extracted from the included studies concerning the interface with other clinical services. These are represented as quotations that preserve the original wording used by the study authors. It should be stressed that several attempts at grouping the findings were attempted before they appeared coherent.

**Table 2:** Organisation and interface with other services (after Table S5)

Finding group	Descriptive data and participant quotes	Conflicting data and participant quotes	Interpretive findings/reading	Synthetic interpretation
Clinical issues, including clinical information	‘The resident's baseline appeared to be not documented well, updated regularly or readily accessible to clinicians at the time of diagnosis’.^36^	‘More objective information’^25^ resulting in ‘Staff empowerment’^6^ versus ‘You know in your gut that this woman has a UTI’.^25^	Contested clinical knowledge of signs and symptoms	Clinical credibility of information
				Used for IPC
	‘Lack … (or delayed information) about whether a patient is MRSA positive’^27^; ‘Telephone prescriptions not being issued in a timely fashion; causing delays in antibiotics’^27^; ‘lack of timely access to medical information pertinent to appropriate diagnosis and management of UTI’^36^; ‘Lack of onsite doctors to provide immediate clinical assessment’^29^; ‘Delays and difficulties in obtaining… [diagnostic] services meant antibiotics were prescribed empirically’^28^ (and^36^); ‘Lack of access to a physician and pharmacist outside of regular hours’^36^; ‘None of the homes had any access to specialist dermatological support and all relied on GP diagnoses of scabies’.^32^	‘Diagnostic tools… were not used for any [scabies] cases’^32^; ‘Antibiotic prescriptions were made without formal evidence and guidelines due to no specific local antimicrobial policies’.^29^		Information as a scarce commodity
				Clinical hierarchy/specialisms
	‘Disagreement with external organisations and interinstitutional regulations’^35^; ‘Managers felt that GPs were reluctant to visit and prescribe for scabies outbreaks’^32^; ‘Having different GPs complicated the [mass treatment] Process’.^32^	‘Formal medication reviews… only performed annually’.^29^		Reliance on external actors/agencies who are untimely or reluctant to get involved
		‘… In certain peoples' mind they may not be swayed unless they hear it from a physician’.^36^		It is all off‐site

Abbreviations: IPC, Infection Prevention and Control; UTI, urinary tract infection.

Another column (‘conflicting data’) presents interview material that somehow countermanded the bulk of the data found. Comparison of data in this way can be useful in a so‐called ‘refutational synthesis’,^17^ where findings from different studies appear to be contradictory, although that was not the case in this meta‐ethnography. However, comparison of the data in this way within each ‘translation’ (i.e., group of findings in Supporting Information Tables) helped derive the second‐order ‘interpretative findings’ of the raw data. In a good qualitative study, such interpretations will be found in the original study reports, but where authors adopt a more descriptive approach, they come from comparison of findings within translations during the synthesis process. Accordingly, this column includes both. Finally, the right‐hand column contains the higher conceptual interpretations, which were made by constant comparison of the contents of the rest of the table.

Turning to the content of Table 2, many first‐order findings focused on the absence of clinical information relevant to IPC or difficulties obtaining it due to record‐keeping or data management systems. Other findings centred on the fact that the necessary information was often located in another organisational entity, such as the hospital pharmacy. Those actors who were needed to formulate diagnoses and treatments, such as GPs, were not on hand and obtaining a diagnosis could present challenges for the priorities of staff on the ground. These issues led to delays in obtaining diagnostic information or treatments. Conflicting findings pointed to a distinction between the real‐world intelligence of care home staff and clinicians who could at times be apparently sceptical about the clinical skills of care home staff or the need for treatment. A consequence of this was that treatment could be delivered in the absence of a relevant diagnosis, for example, by a possibly harassed locum doctor operating out of hours. This goes against the principles of IPC, especially in relation to the issue of antibiotic resistance.

Translating the findings into one another led to the interpretations that clinical knowledge in IPC is a contested area that can lead to questions about the credibility of information related to signs and symptoms. The information necessary to enact IPC is hard to come by (‘a scarce commodity’) and there is reliance on health workers located beyond the control of the care home. Ultimately, the tools necessary for the timely enactment of IPC are ‘all off‐site’.

### 3.3 Outcome of translation

An interpretive reading of the completed theme group tables revealed certain domains of concern, including a perceived low‐skills base in care assistant staff and a lack of effective monitoring or surveillance systems (Table S2); limits to IPC practice in the care home environment (Table S4); and diagnostic and management conflicts between offsite GPs, for example, and care staff who were perceived to lack training or competence (Table S5).

### 3.4 Outcome of synthesis process

In terms of explaining IPC practice, the studies largely distinguished between nurses and nursing assistants; between care home staff and medical staff or services; between residents and their staff carers; or between care staff and other staff not involved in face‐to‐face personal care. A few studies appeared to perceive that poor IPC practice was due to subordinate and poorly paid staff.^9^, ^28^, ^33^, ^35^ Although the use of such staff appeared ubiquitous across the studies, it appears as an essential reality of current nursing and residential care home provision.

**Figure 2:**
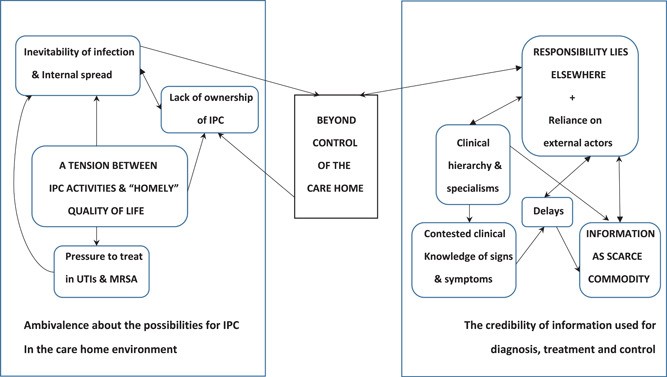
IPC lies outside the control of the care home. IPC, Infection Prevention and Control

Where concepts have been developed that go beyond the findings of the original studies, by the process described above, the text is shown in blue (Tables S2–S5). It was found that these concepts could be related as a theory of IPC in care homes. The main issues are encapsulated in Figure 2, where it can be seen that the control of IPC is understood to lie outside the nursing or residential care home.

There were two separate issues at play in viewing the control of IPC as something to lie outside the nursing or residential care home (left‐hand side of the diagram in Figure 2). One issue concerns a perception that the spread of infections in a care home environment is inevitable, and by extension, results in high ambivalence among staff members about the need for and benefits of applying IPC measures in nursing or residential care homes. A more fundamental issue relates to the tension between IPC practice (e.g., gowns, gloves and resident isolation) and resident quality of life (as in freedom to move around the facility and interact with other residents and staff). The revelation that IPC may be limited to a resident's room, and yet said resident is still free to use communal areas of the home, points to the potential for IPC to be seen as an act, or ritual, as opposed to an effective means of containing the spread of infections. Another example of a tension with quality of life was when a care assistant or night‐time locum doctor is convinced that a resident is displaying symptoms of a urinary tract infection and may feel pressured into securing treatment in the absence of a confirmed diagnosis, for example, due to representations from family members. Moreover, nursing or residential care home staff rarely perceive that they contribute and have ownership of IPC measures, which amplifies perceptions that IPC is something that metaphorically lies beyond the nursing or residential care home.

The right‐hand box in Figure 2 presents a different group of issues concerning the availability or credibility of information critical to patient care including diagnosis, treatment and control. One major problem is that information in relation to IPC is both hard to come by and at times is actively challenged due to communication failures or hierarchical issues. So far as the care home is concerned, all the clinical resources they need are off site and potentially without control or influence.

**Figure 3:**
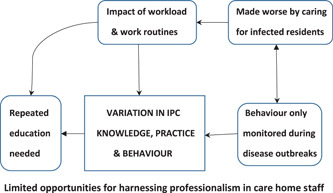
Variation in staff attitudes, knowledge and behaviour

A smaller group of issues that did not fit in the synthesized concepts captured in Figure 2 formed a separate set of relationships that explained the variation in staff knowledge and behaviour related to IPC (Figure 3). Although staff training and education could help improve IPC in nursing and residential care homes, staff in these services have limited opportunities to harness professionalism. A negative feedback loop or vicious cycle is formed, whereby workload and education issues known to affect IPC are themselves further impacted by dealing with the additional challenges of an infection breakout.

## 4 DISCUSSION

### 4.1 Summary of findings

The main finding from this review of IPC in residential and nursing homes was that infection control was revealed as something seen to lie conceptually ‘outside’ the control of the care home, whether according to the origins of the infection, or responsibility for control measures. Translating findings found on these matters yielded further insights that went beyond those seen in the studies. Perhaps of most concern is a negative feedback loop, whereby periods of infection breakout actually make IPC behaviour even harder than it is already due to pressures of monitoring and workload. Other examples of deeper insights include questions about the benefits of training staff if adherence to guidelines or desired training outcomes are not properly monitored. A reactive pose was evident in that training and monitoring may not be initiated until after a failed regulatory inspection. Our findings describe situations where staff afforded ‘low‐skills’ status are then reliant upon communication with a system of external ‘high‐skills’ experts to accomplish clinical work.

Considering the barriers and facilitators to enacting IPC practice in care homes that our study has identified, it is evident that many IPC practices (including isolation or wearing gowns and gloves) can be viewed as antithetical to notions of a homely environment. In IPC practice in the care home, a distinction appeared between what is possible in a resident's room versus what is possible in other areas of the home. In everyday care, staff had to cope with pressures (e.g., prescribing antibiotics before obtaining a lab result) and moral dilemmas of enacting ‘efficiency thoroughness’ trade‐offs.^38^

By bringing together findings from staff and resident participant groups, both appeared ambivalent regarding IPC in the care home settings. It was interesting that just as infections were often seen as an external issue, according to the theory presented herein, so individual residents might view IPC as ‘somebody else's problem’. Essentially, IPC was difficult in everyday practice due to the needs of diverse residents and the social organisation of the care homes.

### 4.2 Relation to the wider literature

Working in a residential or nursing home for older people can be a stressful occupation. This may precipitate burnout,^39^ which this meta‐ethnography found to be a limiting factor in effective IPC. A questionnaire study identified perceived low wages, plus a shortage of staff and resources as contributory factors.^40^ In an environment where aspects of the job are demanding, and people can feel they lack managerial support, some may ‘be happy to let colleagues do the work’.^40^ This is of concern, given the importance of organisational culture in patient safety in general^41^ and in realizing effective IPC practice in particular.^42^ A comparative study of frontline care workers in Canada and Scandinavia went so far as to suggest that organisational factors in care home settings set the context for ‘structural violence’, usually experienced by staff from residents and sometimes on a daily basis.^43^ In another publication from the same study, geographical differences in the experience of care staff were explained by different models of care: ‘highly differentiated task‐oriented work’ (Canada, higher levels of violence against staff) versus an ‘integrated relational care work model’ (Sweden, lower levels of violence).^44^ These issues present challenges for the enactment of IPC in different residential care settings and may go some way towards explaining some of the findings in this meta‐ethnography.

In other studies, violence or abuse towards care home residents has also been linked to the organisation of care.^45^ While the issue of abuse may appear tangential to the focus of this review, it would not be a stretch to argue that deficiencies in IPC could be seen to constitute a form of abuse and IPC is unarguably part and parcel of care quality. More importantly, the kinds of factors found to reduce the incidence of abuse, such as working on the professional development of staff and improving their morale and confidence,^45^ are also likely to be effective in improving IPC practice in care homes. While it may seem intuitive to think in terms of education and training, a review found that education alone is insufficient and needs to be grounded in raising the status of care homes and adopting a relationship‐centred approach to IPC,^46^ perhaps like that found in Sweden.^44^

### 4.3 Strengths, limitations and reflexivity

The strengths of this study included the fact that independent reliability checks were performed during the searches, published reporting standards^18^ were used and that the synthesis resulted in second‐ and third‐order concepts from the primary studies. Meta‐ethnography is a form of primary qualitative data analysis applied to reports of qualitative studies.^17^ As such, it is interpretive, which means that teams with different interests could yield different results. The involvement of a large, multidisciplinary team has been a strength in this study. The studies spoke to similar issues, and it was relatively straightforward to organize the results for analysis purposes. It was perhaps surprising that the same study results seemed to apply in all national contexts, although 13/15 described reports from the United Kingdom or North America. It is likely that studies based in non‐Western countries or low‐ and middle‐income countries would yield a different pattern of findings in terms of enacting IPC in nursing and residential care homes for older people. Moreover, no relevant studies pertaining specifically to Covid‐19 have been included in this meta‐synthesis because no such studies were available at the time the searches were undertaken. Hence, these findings would represent practice on the ground before the start of the Covid‐19 pandemic.

### 4.4 Implications for practitioners and policy makers

One major implication highlighted in this study is the importance of care homes implementing WHO recommendations on IPC.^11^ Training of staff is necessary but not sufficient to improve IPC practice in nursing and residential care homes. Training needs to be embedded within a coherent programme also including guidelines, monitoring and testing. Overall, the meta‐synthesis points to the utility of upgrading health care assistants to enhance their clinical responsibilities. This would require significant investment and might be unworkable within the current model of service provision. A conclusion is that IPC is not something that can be attended to in isolation; it requires wholesale attention to fundamental issues in the organisation and delivery of services.

Several studies appear to attribute responsibility for poor IPC to care assistants, nursing staff or GPs for deficiencies in IPC in care homes. This is of concern, given the ways in which the Covid‐19 pandemic has shone a light on the largely marginalized status of the care home workforce.^47^ A Swedish study found that healthcare assistants in long‐term care facilities could detect early signs of infection,^48^ and ways might be explored to better harness such professional skills for the furtherance of IPC in residential care settings for older people.

Another implication of the findings is the need to alter staff perceptions that infections may be seen as inevitable in residential care settings. The reasons underpinning these perceptions of infection inevitability and IPC pointlessness in care homes are unclear, but may simply reflect previous negative experiences with IPC in these settings. Behavioural science, organisational support and better safety climate could help towards challenging those perceptions that could act as barriers in implementing sustainable IPC improvements in nursing and residential care homes.

## 5 CONCLUSION

The Covid‐19 pandemic is likely to have had a significant impact on the enactment of IPC in care homes. The findings of this study represent IPC practice before the start of the pandemic, but they will be useful for those examining IPC behaviour in care homes during the Covid‐19 pandemic and subsequently.

## AUTHOR CONTRIBUTIONS

Gavin Daker‐White designed the study and led on literature searches, data extraction, analysis and drafting the manuscript. Maria Panagioti, Sally Giles, Tom Blakeman, Victoria Moore, Alex Hall, Paul P. Jones, Oliver Wright, Bethany Shears, Natasha Tyler and Stephen Campbell contributed to data extraction, quality appraisal, analysis and drafting of the manuscript.

## CONFLICT OF INTERESTS

The authors declare that there are no conflict of interests.

## Supporting information

Supporting information.

## Data Availability

All the data are contained in either the manuscript or in the associated Supporting Information Tables.

## References

[1] Comas‐Herrera A , Zalakaín J , Litwin C , Hsu AT , Lane N , Fernández JL . Mortality associated with COVID‐19 outbreaks in care homes: early international evidence. LTCcovid.org, International Long‐Term Care Policy Network, CPEC‐LSE; 2020.

[2] Daly M . COVID‐19 and care homes in England: what happened and why? Soc Pol Admin. 2020;54(7):985‐998. 10.1111/spol.12645 PMC7461496 32904948

[3] Iacobucci G . Covid‐19: lack of PPE in care homes is risking spread of virus, leaders warn. BMJ. 2020;368:m1280. 32220878 10.1136/bmj.m1280

[4] Hammerschmidt J , Manser T . Nurses' knowledge, behaviour and compliance concerning hand hygiene in nursing homes: a cross‐sectional mixed‐methods study. BMC Health Serv Res. 2019;19(1):547. 31382968 10.1186/s12913-019-4347-z PMC6683349

[5] Stone PW , Chastain AM , Dorritie R , et al. The expansion of National Healthcare Safety Network enrollment and reporting in nursing homes: lessons learned from a national qualitative study. Am J Infect Control. 2019;47(6):615‐622. 30850253 10.1016/j.ajic.2019.02.005 PMC6544481

[6] Krein SL , Harrod M , Collier S , et al. A national collaborative approach to reduce catheter‐associated urinary tract infections in nursing homes: a qualitative assessment. Am J Infect Control. 2017;45(12):1342‐1348. 28807424 10.1016/j.ajic.2017.07.006 PMC5828510

[7] Moretti F , Visentin D , Bovolenta E , et al. Attitudes of nursing home staff towards influenza vaccination: opinions and factors influencing hesitancy. Int J Environ Res Public Health. 2020;17(6):1851. 10.3390/ijerph17061851 PMC7143910 32178426

[8] Elias C , Fournier A , Vasiliu A , et al. Seasonal influenza vaccination coverage and its determinants among nursing homes personnel in western France. BMC Public Health. 2017;17(1):634. 28687075 10.1186/s12889-017-4556-5 PMC5501011

[9] Travers J , Herzig CT , Pogorzelska‐Maziarz M , et al. Perceived barriers to Infection Prevention and Control for nursing home certified nursing assistants: a qualitative study. Geriatr Nurs. 2015;36:355‐360. 26071320 10.1016/j.gerinurse.2015.05.001 PMC4600411

[10] Nace DA , Perera S , Handler SM , Muder R , Hoffman EL . Increasing influenza and pneumococcal immunization rates in a nursing home network. J Am Med Dir Assoc. 2011;12(9):678‐684. 21450182 10.1016/j.jamda.2010.05.002 PMC4893952

[11] World Health Organization . Improving Infection Prevention and Control at the health facility: interim practical manual supporting implementation of the WHO Guidelines on Core Components of Infection Prevention and Control Programmes; 2018.

[12] Lee MH , Lee GA , Lee SH , Park YH . Effectiveness and core components of Infection Prevention and Control programmes in long‐term care facilities: a systematic review. J Hosp Infect. 2019;102:377‐393. 30794854 10.1016/j.jhin.2019.02.008

[13] Eveillard M , Raymond F , Guilloteau V , et al. Impact of a multi‐faceted training intervention on the improvement of hand hygiene and gloving practices in four healthcare settings including nursing homes, acute‐care geriatric wards and physical rehabilitation units. J Clin Nurs. 2011;20(19‐20):2744‐2751. 21366742 10.1111/j.1365-2702.2011.03704.x

[14] Andersson H , Lindholm C , Iversen A , et al. Prevalence of antibiotic‐resistant bacteria in residents of nursing homes in a Swedish municipality: healthcare staff knowledge of and adherence to principles of basic infection prevention. Scand J Infect Dis. 2012;44(9):641‐649. 22680834 10.3109/00365548.2012.671956

[15] Care Quality Commission . Working together to prevent and control infections: a study of the arrangements for Infection Prevention and Control between hospitals and care homes; 2009.

[16] Health Protection Agency A report on the management of diarrhoea in care homes: [Including an assessment of the implications for recognition and management of residents with Clostridium difficile infectin; 2010. https://assets.publishing.service.gov.uk/government/uploads/system/uploads/attachment_data/file/360128/FINAL_REPORT_diarrhoea_in_care_homes_10_August_2010.pdf. Accessed December 17, 2020.

[17] Noblit GW , Hare RD . Meta‐ethnography: Synthesizing Qualitative Studies. Sage; 1988.

[18] France EF , Cunningham M , Ring N , et al. Improving reporting of meta‐ethnography: the eMERGe reporting guidance. BMC Med Res Methodol. 2019;19:25. 10.1186/s12874-018-0600-0 30709371 PMC6359764

[19] Alldred DP , Kennedy MC , Hughes C , et al. Interventions to optimise prescribing for older people in care homes. Cochrane Database Syst Rev. 2016;2:CD009095. 10.1002/14651858.CD009095.pub3 26866421 PMC7111425

[20] Wang J , Liu F , Tan JBX , Harbarth S , Pittet D , Zingg W . Implementation of Infection Prevention and Control in acute care hospitals in Mainland China—a systematic review. Antimicrob Resist Infect Control. 2019;8:32. 10.1186/s13756-019-0481-y 30792854 PMC6371478

[21] McKibbon KA , Wilczynski NL , Haynes RB . Developing optimal search strategies for retrieving qualitative studies in PsycINFO. Eval Health Prof. 2006;29(4):440‐454. 17102065 10.1177/0163278706293400

[22] Daker‐White G , Hays R , McSharry J , et al. Blame the patient, blame the doctor or blame the system? A meta‐synthesis of qualitative studies of patient safety in primary care. PLoS One. 2015;10(8):e0128329. 26244494 10.1371/journal.pone.0128329 PMC4526558

[23] Dixon‐Woods M , Cavers D , Agarwal S , et al. Conducting a critical interpretive synthesis of the literature on access to healthcare by vulnerable groups. BMC Med Res Methodol. 2006;6:35. 16872487 10.1186/1471-2288-6-35 PMC1559637

[24] Campbell R , Pound P , Morgan M , et al. Evaluating meta‐ethnography: systematic analysis and synthesis of qualitative research. Health Technol Assess. 2011;15(43):1‐164. 10.3310/hta15430 22176717

[25] Lohfeld L , Loeb M , Brazil K . Evidence‐based clinical pathways to manage urinary tract infections in long‐term care facilities: a qualitative case study describing administrator and nursing staff views. J Am Med Dir Assoc. 2007;8:477‐484. 17845952 10.1016/j.jamda.2007.05.006

[26] Hutt E , Radcliff TA , Oman KS , et al. Impact of NHAP guideline implementation intervention on staff and resident vaccination rates. J Am Med Dir Assoc. 2010;11:365‐370. 20511104 10.1016/j.jamda.2009.09.017

[27] Morrow E , Griffiths P , Rao GG , Flaxman D . “Somebody else's problem?” Staff perceptions of the sources and control of meticillin‐resistant *Staphylococcus aureus* . Am J Infect Control. 2011;39:284‐291. 21030114 10.1016/j.ajic.2010.06.018

[28] McClean P , Tunney M , Parsons C , Gilpin D , Baldwin N , Hughes C . Infection control and meticillin‐resistant *Staphylococcus aureus* decolonization: the perspective of nursing home staff. J Hosp Infect. 2012;81:264‐269. 22727826 10.1016/j.jhin.2012.05.005

[29] Lim CJ , Kwong MW , Stuart RL , et al. Antibiotic prescribing practice in residential aged care facilities‐health care providers' perspectives. Med J Aust. 2014;201:101‐105. 10.5694/mja13.00102 25045989

[30] Cohen C , Pogorzelska‐Maziarz M , Herzig CTA , et al. Isolation‐based Infection Prevention and Control practices in nursing homes: a qualitative study. BMJ Qual Saf. 2015;24(10):630‐636. 10.1136/bmjqs-2015-003952 PMC4575834 26002947

[31] Travers J , Herzig CT , Pogorzelska‐Maziarz M , et al. Understanding Infection Prevention and Control in nursing homes: a qualitative study. Geriatr Nurs. 2015;36:267‐272. 25794923 10.1016/j.gerinurse.2015.02.023 PMC4530090

[32] Hewitt KA , Nalabanda A , Cassell JA . Scabies outbreaks in residential care homes: factors associated with late recognition, burden and impact. A mixed methods study in England. Epidemiol Infect. 2015;143:1542‐1551. 25195595 10.1017/S0950268814002143 PMC9507193

[33] Albrecht JS , Croft L , Morgan DJ , Roghmann MC . Perceptions of gown and glove use to prevent methicillin‐resistant *Staphylococcus aureus* transmission in nursing homes. J Am Med Dir Assoc. 2017;2017(18):158‐161. 10.1016/j.jamda.2016.08.016 PMC5272866 27687079

[34] Morales K . *Testing the effect of a resident‐focused hand hygiene intervention in a long‐term care facility: a mixed methods feasibility study*. Doctoral dissertation. Mercer University; 2017.

[35] Lee CY , Lee MH , Lee SH . Nurses' views on infection control in long‐term care facilities in South Korea: a focus group study. Korean J Adult Nurs. 2018;30:634‐642.

[36] Chan AJ . *A qualitative study on perceived barriers and facilitators of implementing an antimicrobial stewardship intervention in the management of urinary tract infections in a long‐term care setting*. Masters Dissertation. McMaster University; 2019.

[37] Van Tiem JM , Friberg JE , Cunningham Goedken C , et al. Environmental service workers as potential designers of infection control policy in long‐term care settings. Am J Infect Control. 2020;48:398‐402. 32087975 10.1016/j.ajic.2020.01.014

[38] McNab D , McKay J , Shorrock S , Luty S , Bowie P . Development and application of ‘systems thinking’ principles for quality improvement. BMJ Open Qual. 2020;9:e000714. 10.1136/bmjoq-2019-000714 PMC7103793 32209593

[39] Cooper SL , Carleton HL , Chamberlain SA , Cummings GG , Bambrick W , Estabrooks CA . Burnout in the nursing home health care aide: a systematic review. Burnout Res. 2016;3(3):76‐87.

[40] Dunn LA , Rout U , Carson J , Ritter SA . Occupational stress amongst care staff working in nursing homes: an empirical investigation. J Clin Nurs. 1994;3(3):177‐183. 7834131 10.1111/j.1365-2702.1994.tb00383.x

[41] Kaufman G , McCaughan D . The effect of organisational culture on patient safety. Nurs Stand. 2013;27(43):50‐56. 10.7748/ns2013.06.27.43.50.e7280 23987721

[42] Borg MA . Cultural determinants of infection control behaviour: understanding drivers and implementing effective change. J Hosp Infect. 2014;20186(3):161‐168. 10.1016/j.jhin.2013.12.006 24534705

[43] Banerjee A , Daly T , Armstrong P , Szebehely M , Armstrong H , Lafrance S . Structural violence in long‐term, residential care for older people: comparing Canada and Scandinavia. Soc Sci Med. 2012;74(3):390‐398. 22204839 10.1016/j.socscimed.2011.10.037 PMC4069106

[44] Daly T , Szebehely M . Unheard voices, unmapped terrain: care work in long‐term residential care for older people in Canada and Sweden. Int J Soc Welf. 2012;21(2):139‐148. 24999303 10.1111/j.1468-2397.2011.00806.x PMC4081477

[45] Lawrence V , Banerjee S . Improving care in care homes: a qualitative evaluation of the Croydon care home support team. Aging Ment Health. 2010;14(4):416‐424. 20455117 10.1080/13607860903586144

[46] Nolan M , Davies S , Brown J , et al. The role of education and training in achieving change in care homes: a literature review. J Res Nurs. 2008;13(5):411‐433.

[47] McGilton KS , Escrig‐Pinol A , Gordon A , et al. Uncovering the devaluation of nursing home staff during COVID‐19: are we fuelling the next health care crisis? J Am Med Dir Assoc. 2020;21(7):962‐965. 32674829 10.1016/j.jamda.2020.06.010 PMC7287421

[48] Tingström P , Milberg A , Sund‐Levander M . Early nonspecific signs and symptoms of infection in institutionalized elderly persons: perceptions of nursing assistants. Scand J Caring Sci. 2010;24(1):24‐31. 19954493 10.1111/j.1471-6712.2008.00680.x

