# Supplementary material for: Beyond the control of the care home: A meta‐ethnography of qualitative studies of Infection Prevention and Control in residential and nursing homes for older people
Source: Health Expect. 2021 Aug 21;25(5):2095–106. doi: 10.1111/hex.13349 (PMC9615085; doi:10.1111/hex.13349)
Supplement: Supplementary file 1 — Supporting information. [file HEX-25--s001.docx]

**eTable 1(a): Searches**

| **Database: Embase <1980 to 2020 Week 19>, Ovid MEDLINE(R) <1946 to April Week 5 2020>, APA PsycInfo <1806 to May Week 2 2020>** | |
| --- | --- |
| 1 | homes for the aged/ or "homes for the aged".tw. (22651) |
| 2 | exp nursing homes/ or nursing home?.tw. (117093) |
| 3 | (aged adj2 (care or nursing or healthcare or residential) adj2 (facility or facilities or home?)).ti,ab. (3138) |
| 4 | ((geriatric or elderly) adj2 (facility or facilities or care home?)).ti,ab. (1272) |
| 5 | hospitals, veterans/ (37660) |
| 6 | ((care or convalescent) adj (home? or center? or centre? or facility or facilities)).ti,ab. (134573) |
| 7 | ((skilled or intermediate) adj (nursing facility or nursing facilities)).ti,ab. (6988) |
| 8 | (resident* adj2 (care or facility or facilities)).ti,ab. (28234) |
| 9 | ((nursing or group or residential) adj home?).ti,ab. (79594) |
| 10 | long-term care/ (156269) |
| 11 | ((longterm or long term) adj3 (care or facility or facilities)).ti,ab. (61085) |
| 12 | (healthcare adj2 (facility or facilities)).ti,ab. (11789) |
| 13 | residential facilities/ (11524) |
| 14 | assisted living facilities/ (3725) |
| 15 | assisted living.ti,ab. (5678) |
| 16 | halfway houses/ (2151) |
| 17 | or/1-16 (480498) |
| 18 | exp aged/ (5989373) |
| 19 | geriatrics/ (67227) |
| 20 | (gerontol* or ageing or aging or elder* or geriatric* or seniors or old age or older or late* life).ti,ab. (2059516) |
| 21 | (older adj (person* or people or adult* or patient* or inpatient* or outpatient*)).ti,ab. (370791) |
| 22 | veterans/ (48362) |
| 23 | veteran*.ti,ab. (96470) |
| 24 | or/18-23 (7207637) |
| 25 | hospital infection.mp. (45678) |
| 26 | cross infection.mp. (76607) |
| 27 | infection control.mp. (129547) |
| 28 | infection prevention.mp. (66841) |
| 29 | (infection and control).mp. [mp=ti, ab, hw, tn, ot, dm, mf, dv, kw, fx, dq, nm, kf, ox, px, rx, ui, sy, tc, id, tm, mh] (626885) |
| 30 | nosocomial infection.mp. (15007) |
| 31 | healthcare associated infection.mp. (6331) |
| 32 | catheter related infection.mp. (2378) |
| 33 | catheterization.mp. (306744) |
| 34 | catheter infection.mp. (18498) |
| 35 | catheter associated infection.mp. [mp=ti, ab, hw, tn, ot, dm, mf, dv, kw, fx, dq, nm, kf, ox, px, rx, ui, sy, tc, id, tm, mh] (462) |
| 36 | methicillin resistant staphylococcus aureus.mp. (78551) |
| 37 | clostridium difficile.mp. [mp=ti, ab, hw, tn, ot, dm, mf, dv, kw, fx, dq, nm, kf, ox, px, rx, ui, sy, tc, id, tm, mh] (41798) |
| 38 | vancomycin resistant enterococcus.mp. [mp=ti, ab, hw, tn, ot, dm, mf, dv, kw, fx, dq, nm, kf, ox, px, rx, ui, sy, tc, id, tm, mh] (8937) |
| 39 | vancomycin resistant enterococci.mp. (6606) |
| 40 | bacteremia.mp. (87773) |
| 41 | pneumonia, ventilator associated.mp. (3796) |
| 42 | hand washing.mp. (16789) |
| 43 | hand disinfection.mp. (6835) |
| 44 | hand hygiene.mp. (11019) |
| 45 | qualitative.tw. (614958) |
| 46 | interviews.mp. (583233) |
| 47 | themes.tw. (230056) |
| 48 | experience.mp. (1895825) |
| 49 | 17 and 24 (184429) |
| 50 | or/25-44 (1215845) |
| 51 | or/45-48 (2821741) |
| 52 | 49 and 50 and 51 (576) |
| 53 | remove duplicates from 52 (454) |

**eTable 1(b): Data Extraction form: Enacting infection prevention and control (IPC) in elderly long-term care facilities (LTCFs) and transitional care: a meta-synthesis of qualitative studies**

| **Paper ID** |  | | |
| --- | --- | --- | --- |
| Date |  | | |
| Extracted by |  | | |
| Title of Article |  | | |
| Publication Date |  | | |
| Type of article |  | | |
| **Study details** | Location / Country |  | |
|  | Research question / Objectives |  | |
| **Focus** | Within care home, care transfers or both? |  | |
| **Quality** | Are the aims and objectives clearly stated? | | Yes / No |
|  | Is the design clearly specified and appropriate? | | Yes / No |
|  | Do the researchers provide a clear account of the process through which findings were produced? | | Yes / No |
|  | Do the researchers display enough data to support their interpretations and conclusions? | | Yes / No |
|  | Is the method of analysis appropriate and adequately explicated? | | Yes / No |
|  | QUALITY? | Excellent / Acceptable / Unacceptable | |
|  | If ‘Unacceptable,’ Why? |  | |
|  | Is it a “key paper”? If so – why? |  | |
| **Participants** | Population |  | |
|  | Age |  | |
|  | Age (mean/range) |  | |
|  | Gender (M/ F) |  | |
|  | Ethnicity |  | |
|  | Recruitment / sampling (inclusion criteria, response rates, diffs. between responders and non-responders) |  | |
| **Data Collection** | Methods |  | |
|  | Trustworthiness |  | |
| **Data Analysis** | Method |  | |
| Is it primarily descriptive? | |  | |
| Is it an ‘exploratory’ study, pilot or protocol? | |  | |
| How are results presented? | |  | |
| **Main findings**: Theme / Concept #1  Outline in detail, using author’s own words (in quotation marks with page references) in describing main findings. | | If you make your own interpretations of the data/findings, record these but clearly label as your own interpretation | |
| **Main findings**: Theme / Concept #2 | |  | |
| **Main findings**: Theme / Concept #3 | |  | |
| **Main findings**: Theme / Concept #4 | |  | |
| **Main findings**: Theme #5 | |  | |
| **Main findings**: Theme #6 | |  | |
| **Main findings**: Theme #7 | |  | |
| **Main findings**: Theme #8 | |  | |
| **Memos (i.e. implications for developing concepts and theories)** | |  | |
| **Comments (Limitations, reviewer comments, etc.)** | |  | |
| **References** – Possible new | |  | |
| **References** – For Background | |  | |

**eTable 2** - Opportunities & Threats to IPC in elderly residential and nursing homes: Organisation of Care

| **Finding Group** | **Descriptive data and participant quotes** | **Conflicting data and participant quotes** | **Interpretive Findings / Reading** | **Synthetic Interpretation** |
| --- | --- | --- | --- | --- |
| Knowledge, attitude and practice | No disease surveillance [33, 36] or monitoring of IPC practice [30, 36] or outcomes [26]; “lack of nursing record and documentation” [36]; informal compliance monitoring practices [31]  “Still does it the old way” [9]; “The concept of effective containment varied” [30]; “The response from GPs was inconsistent” [32]; “Complacency” around IPC: “not performed properly by some staff members because of their attitude” [28]  “Staff had not previously thought about protecting themselves from MRSA” [33]; “Lack of leadership” [36]; “Poor”/“lack” understanding [33, 30]; “That’s not well-understood, but hand-washing is” [9]. Tool developed “was not effective because some … could not read it”; “If you come from a culture where you don’t really discuss medical issues … that could be a barrier.” [9]  “Obtaining cooperation from all departments” [31]; “Unclear separation of duties between nurses and unlicensed-HCP” [36]  **On perceived sources of infection:**  “The hospital blames us and we blame them”; “From care homes” [27]; Hospitals seen as a source of infections and resistant bacteria [28]; Patient characteristics; hospital transport or transitions (also [29]); relative’s IPC practice; staff knowledge; staff laziness; systems issues; trends in antibiotic prescribing; places of close contact [27]; “‘’Self’’ and ‘‘team’’ causes of MRSA were described as ‘‘inevitable’’ …or… attributed to ‘‘lapses’’ because of understaffing or the impracticalities” [27]  **Education Focus**:  Continual education programs needed because of staff turnover [26, 35]; “Some of them are part-time and some of them work shifts” [25]; “limited opportunities to educate this group [CNAs] on IPC practices” [9]; “Lower educational requirements of CNAs” [9]  “[CNAs] learned the procedure, but not … the why” [9]; “‘Using gowns and gloves primarily as self-protection against contact with bodily fluids, not MRSA” [33]; “Lack of knowledge regarding key IPC concepts among care workers … were perceived to impede nurses’ efficacy in adhering to IPC roles” [36]; “Surveillance revealed unacceptable practice patterns in IPC” blamed largely on hourly paid staff [9]; “Mixed … views on whether nurse review of infections aided to guide decisions regarding antibiotics due to differences in nursing grade and experience” [29] | “When outbreaks occur, the infection prevention behaviors of nurses and ESWs are most often scrutinized.” [38]  “The way we do things around here” [28]; Staff team views “about what is normal and acceptable infection control” [27]; | A distinction between team & individual perceptions or behaviour  Note othering of CNAs  “The significance of long-held beliefs in influencing risk perception” [27]  Are the right people receiving the IPC training?  Cultural issues  Low skills base | **No IPC data**  **Behaviour only scrutinised during disease outbreaks**  **Variation in IPC knowledge, practice and behaviour**  **Unclear care boundaries**  **Beyond the control of the care home [CORE CONCEPT] (See also Tables 3 & 4)**  **A need for repetitive re-enforcement of IPC education messages**  **Staff grades**  **? Clinical ‘One-up-manship’** |
| Workload / Lack of Time or resource | “When the workload was unmanageable” [28] / “Being in a hurry” [30] / “Lack of time” [37] impacted IPC practice  “More time and other resources that enabled communication” [30]; “Infrequent work schedules of per-diem and part-time staff posed difficulties for IPC communication” [9]; “Challenges in communication and cooperation among HCP” [36]; “I’d like to see my nurse’s aide communicate … more” [9]  “The availability of private rooms” [30]; “Applying the lotion to, and showering, all residents on the same day was logistically difficult” [in scabies ‘mass treatment’] [32]; “Logistical barriers” [35]; “Incorrect prescribing increased costs” [of scabies treatment] [32] | “Variation in delivery of wipes appeared to be related to the work routine of the staff.” [34]  “Increased workload due to required [PPE] when caring for residents with [HAIs]” [34]  “Working with what you have” [30]; Control measures implemented only … “after regulatory inspections” [28] and [31] |  | **IPC Practice impacted by workload and work routine; made worse by caring for infected residents**  **Resourcing** |

**eTable 3: Barriers and facilitators to enacting IPC in care homes: Individual behaviour and institutional goals**

| **Finding Group** | **Descriptive data and participant quotes** | **Conflicting data and participant quotes** | **Interpretive Findings / Reading** | **Synthetic Interpretation** |
| --- | --- | --- | --- | --- |
| Problems getting staff to enact IPC procedures | “Lack of initial buy-in from nurses”; “difficulties getting physicians to accept and follow the protocol” (“conflicting views” in [28]); “ensuring that part-time or new staff correctly followed procedures” [25]  “Issues with locum doctors having lower threshold for antibiotic prescriptions and overtreating … Pressure of overnight decisions and fear of not adding patients’ management plans” [29]; “GPs and nurses were more likely to prescribe antibiotics (especially broad-spectrum) in those with higher frailty scores and cognitive impairment due to difficulties in obtaining patient histories, samples and higher risk of rapid decline. This is not based on evidence based practice.” [29]  “GPs also felt pressure to overtreat by the nursing staff- ‘we do get pressured a little bit by nursing staff to do something’” [29]  “faced opposition from residents’ families” [25]; “family pressure” [37] | Staff tend to attribute successes in infection control to staff performance such as awareness and adherence to practice guidance” [27]  “Collaborating with residents and their families to create care plans for residents can partially address the barrier of family pressure” [37] | **“Lack of ownership of IPC created breakdown in infection control practices and communication” [9]** links with “**Beyond the control of the care home**” (Table 2, perceived sources of infection) | **Resistance to protocols or new ways of working**  **Contextual circumstances when antibiotics are prescribed – Linked to “Staff grades” (Table 2)**  **Pressure to Treat (in UTIs & MRSA)** |
| Staff motivations “for practicing good infection control” [27] | “The complexity of the residents’ needs;” “tensions between the facility being the residents’ home and the need for IPC”; “The importance of resident quality of life and concerns that isolation practices conflicted … was pervasive throughout the interviews;” [31] “Isolation seen as ‘horrible’” [30]; “Conflict in maintaining an environment that was both ‘homely’ and clinical” [28]  “Both inside and outside the room, the “homelike” setting of the CLC disrupts infection prevention and control practices designed for acute care settings. Infection prevention work in rooms requires collaboration with the person living in the room.”; “Resident rooms are a crossroads for infection control” [38]  “Collaboration was most effective when staffing was regular and members have a prior relationship with each other and trust has been built.” [37]  “Annual meetings with per-diem and part-time staff, identifying and in-servicing those staff when they first started working at the facility, and providing one-on-one in- services when feasible. Having more permanent staff, lower turn- over, and an infection control coordinator at the NH facility were described as facilitators to IPC compliance.” [9]; “Using a standard algorithm for UTI diagnosis as well as having a list of commonly used antibiotics for treating UTI were promoted as helpful tools to use in educating clinicians.” [37]  “Personal safety and fear of blame or stigma from the media or … public” [26]; “Interviewees also noted fears of spreading infection among the residents, and to themselves and their families” [29]; Importance of senior staff in promoting IPC [26]; “encouragement from specialist … teams or clinical managers” [27]; Local policy re: informed consent for flu shots [26]  “Wanting to deliver high-quality patient care, willingness to behave in accordance with clear organizational policies”; “Strong relationships with team members in the facility [led to] greater collaboration among all staff participating in the program” [35] | “Sometimes they were able to overcome the tension between resident needs and infection prevention and other times they had to turn admissions away.” [31]  “Targeting infection prevention work in rooms misunderstands how people use the space of the CLC.” [38]  ‘  “Ambiguous guidance on glove use resulting in dependence on knowledge, assessment, and desire for self-protection. For example, staff also reported no glove use for activities involving direct resident contact such as transfers or ambulation” [33] | “It appears that a strong and shared view of good infection control practice (whether or not actual rates are known) supports team collegiality and confidence to challenge poor practice. However, it can lead to a situation in which staff believe they are fulfilling locally defined best-practice, so the risk of MRSA must be coming from elsewhere.” [27] | **A tension between IPC and “homely” quality of life [CORE CONCEPT – See esp Table 4)**  Fear of infection;  **The judgements of outsiders**.  The importance of the policies and behaviour of people further up the hierarchy.  **Harnessing**  **Professionalism** |

**eTable 4: IPC Not seen as appropriate in Care Homes: Lacking organisational responsibility and individual agency**

| **Finding Group** | **Descriptive data and participant quotes** | **Conflicting data and participant quotes** | **Interpretive Findings / Reading** | **Synthetic Interpretation** |
| --- | --- | --- | --- | --- |
| IPC not seen as appropriate in care home environment | “Universal gown and glove use … was not supported by staff or administrators, who expressed concern about staff objections, burden, and compliance, as well as the perception of the community.” [33]; “Were concerned about patient perceptions of gowns” [33]; “I don’t think the facility is going to like us to have gowns on all the time.” [33]  “GPs did not support MRSA decolonization in nursing homes”; “Decolonization in nursing homes was tempered by the risk of recolonization, particularly from hospital admissions” [28]  “Several of the index cases were described as ‘wanderers’ and staff were unsure how to prevent further transmission within the home” [9]; “Difficult to achieve good infection control practices with confused residents, some families, GPs and members of staff who were resistant to change.” [28]  ‘ “It comes from us. Patient to patient, room to room. MRSA is everywhere … and that’s how they get it.” Residents themselves contribute to the spread of MRSA, “People come out of the rooms and they’re touching here and there, even those on isolation. Even though we’ve got residents on isolation, therapy is still going to come and get those people.” ’ [33]  Non-personal care staff did not see IPC as their concern [28]  “Workload;” “time constraints and understaffing impeded effective IPC practice”; “multitasking” [9]; “Mass treatment [in scabies] was a significant extra workload, and most homes needed extra staff” [32]; Problems adding “burdensome” tasks to existing lists of tasks [35] | However, once residents were informed about the purpose of the gown, they were pragmatic, “Now that I know what it’s for, I think it’s very practical.” ’ [33]  “Family noncompliance with resident visitation regulations; High workload caused by great turnover and infection breakout; A need for improvement in staffing and employee support”; “We can only control them when there is a national epidemic event ” [36]  “All staff have a role to play in prevention” [35] | “Some GPs felt national guidelines were not applicable to the patient cohort in the residential care facility” [29] | **Ambivalence about the possibilities for IPC in a care home environment**  **Inevitability of infection and spread – Except in extremis** |
| Residents’ views and IPC behaviour | “Residents felt protected when staff wore gloves, but it was not clear what they felt protected from despite probing the issue. Residents also thought that gloves protected the healthcare worker from contact with bodily fluids.” ’[33]  “Physical limitations, such as arthritis or upper limb amputation as making hand hygiene more” difficult [34]; “Better adherence to hand hygiene in the dining hall when the staff provided a visual and verbal cue, such as: “Here, (name), wipe your hands”;” "I hardly see the residents washing their hands” [34]; “Residents’ ability and willingness to use appropriate personal hygiene, standard precautions and potentially personal protective equipment outside of his/her room” [30]; “A lack of personal accountability for preventing infection in self and others, with few residents speaking in first person” [34]; “Residents viewed others at risk for infection, as sources of infection, and in need of hygiene rather than self. Residents viewed the environment as needing cleaning rather than their own hands as a threat to self” [34]  “The need to educate residents and their families on the risks associated with overprescribing antimicrobials” [25] | “The work that nurses and ESWs do to engage in infection prevention in residents’ rooms is blunted by the reality that residents are generally free to move throughout the facility without wearing PPE themselves” [38] | “Contributes to a broad understanding, among infection control team members, that they “cannot perform [infection control] in the full thrust as we want it in the CLC” [38].  “Responsibility for controlling infections, the resident participants expressed a sense of protection in the long-term care facility which may contribute to a belief that no action is required on the resident's part.” [34] | **IPC Not practically possible due to the organisation of living in the home**  **Responsibility lies elsewhere [CORE CONCEPT]**  **Again – infection as external threat / source “othering” of transmission** |

**eTable 5: Diagnosis, management and treatment largely off site: Organisation and interface with other services.**

| **Finding Group** | **Descriptive data and participant quotes** | **Conflicting data and participant quotes** | **Interpretive Findings / Reading** | **Synthetic Interpretation** |
| --- | --- | --- | --- | --- |
| Clinical Issues, including Clinical Information | “The resident’s baseline appeared to be not documented well, updated regularly or readily accessible to clinicians at the time of diagnosis.” [37]  “A lack … (or delayed information) about whether a patient is MRSA positive is detrimental to … [IPC]” [27]”; “Telephone prescriptions not being issued in a timely fashion; causing delays in antibiotics” [27]; “The lack of timely access to medical information pertinent to appropriate diagnosis and management of UTI” [37]; “Most [scabies] outbreaks were attributed to late diagnosis of the index case;” “Scabies had been misdiagnosed” [32]; “ ‘Lack of onsite doctors to provide immediate clinical assessment’ which lead to antibiotics prescribed empirically with delays in review” [29]; “Delays and difficulties in obtaining … [diagnostic] services meant antibiotics were prescribed empirically for chest and urinary tract infection [28] (and [37]); “Lack of access to a physician and pharmacist outside of regular hours is challenging” [37]; “Sometimes 4 or 6, or maybe 7 hours until we get that antibiotic, they can deteriorate rapidly’” [29]; “None of the homes had any access to specialist dermatological support and all relied on GP diagnoses of scabies” [32];  “Disagreement with external organizations and inter-institutional regulations” [36]; “Managers felt that GPs were reluctant to visit and prescribe for scabies outbreaks. In two outbreaks none of the cases had been visited by a GP, and scabies had been diagnosed by nursing staff” [32]; “Having different GPs complicated the [‘mass treatment’] Process” [32] | “More objective information” [25] resulting in “Staff empowerment” [35] versus “You know in your gut that this woman has a UTI” [25]  “Diagnostic tools … were not used for any [scabies] cases” [32]; “Antibiotic prescriptions were made without formal evidence and guidelines due to no specific local antimicrobial policies” [29]  “Formal medication reviews by senior pharmacists were only performed annually. Although nurses and doctors felt there was a limited role in pharmacists reviewing antibiotic prescriptions.” [29]  “… in certain peoples' mind they may not be swayed unless they hear it from a physician and that is a culture, that's a cultural change just in our society in general” [37] | **Contested clinical knowledge of signs & symptoms** | **The clinical credibility of the information used for diagnosis, treatment and control**  **Information arrives after it was needed**  **Information as a scarce commodity**  **Clinical hierarchy / specialism**  **Reliance on external actors / agencies who are untimely or reluctant to get involved [CORE CONCEPT]**  **It’s all off-site** |
